# Comparison of Surface Markers between Human and Rabbit Mesenchymal Stem Cells

**DOI:** 10.1371/journal.pone.0111390

**Published:** 2014-11-07

**Authors:** Tao-Chen Lee, Tsung-Han Lee, Yu-Hua Huang, Nyuk-Kong Chang, Yu-Jun Lin, Pei-Wen Chang Chien, Wei-Hsun Yang, Martin Hsiu-Chu Lin

**Affiliations:** 1 Department of Neurosurgery, Chiayi Chang Gung Memorial Hospital and Chang Gung University College of Medicine, Chiayi, Taiwan; 2 Department of Neurosurgery, Kaohsiung Chang Gung Memorial Hospital and Chang Gung University College of Medicine, Kaohsiung, Taiwan; 3 Department of Center for Laboratory Animals, Kaohsiung Chang Gung Memorial Hospital and Chang Gung University College of Medicine, Kaohsiung, Taiwan; Shenzhen Institutes of Advanced Technology, China

## Abstract

This study investigated whether there are marked differences in surface markers between rabbit and human mesenchymal stem cells (MSCs). Murine and rabbit MSCs have been reported to be CD90-negative. Rat MSCs have been reported to be CD71-negative. Our previous study also shows that rabbit MSCs are CD29-negative. However, human MSCs are generally considered to be CD29-, CD71-, and CD90-positive. Therefore, the surface markers of human MSCs might differ from those of other species. Rabbit bone marrow MSCs were obtained that had a multi-differentiation potential. The phenotype of these cells was studied using flow cytometry antibodies for 25 rabbit surface markers, namely, CD13, CD14, CD29, CD31, CD34, CD44, CD45, CD49d, CD49f, CD51, CD54, CD59, CD71, CD73, CD90, CD105, CD106, CD133, CD166, MHC I, MHC II, α-smooth muscle actin (α-SMA), cytokeratin, desmin, and vimentin. The phenotype of commercially available human MSCs was similarly studied using antibodies for human surface markers. CD14, CD31, CD34, CD45, CD49d, CD49f, CD51, CD54, CD71, CD106, CD133, MHC II, and cytokeratin were absent from both rabbit and human MSCs, while CD44, α-SMA, and vimentin were present on both cell lines. CD13, CD29, CD59, CD73, CD90, CD105, CD166, and MHC I were present on human MSCs, but not on rabbit MSCs. However, desmin was present on rabbit MSCs, but not on human MSCs. In total, the surface expression of nine markers differed between human and rabbit MSCs, whereas the surface expression of 16 markers was the same in the two cell lines.

## Introduction

Biological and clinical interest in mesenchymal stem cells (MSCs) has increased dramatically over the past two decades [1], [2]. MSCs are multipotent cells that can replicate and have the potential to differentiate into cells of mesenchymal lineages, including bone, cartilage, and fat [2].

According to a report by Pittenger et al., human MSCs are characterized by the presence of particular marker proteins on their surface, including CD29, CD44, CD71, CD90, and CD105, and by the absence of marker proteins of leukocytes and cells of hematopoietic lineage, including CD14, CD34, and CD45 [2]. However, Peister et al. reported that murine MSCs do not express CD90 [3], Lapi et al. reported that rabbit MSCs do not express CD90 [4], and Karaoz et al. reported that rat MSCs do not express CD71 [5]. Our previous study showed that rabbit MSCs are CD29- and CD90-negative [6]. Martínez-Lorenzo et al. found species-related differences in the phenotypes of MSCs from human, rabbit, and sheep [7].

Although this suggests that surface markers are not the same on human MSCs as on MSCs of other species, the number of surface markers studied by Peister et al. and Lapi et al. was relatively small (10 and 11, respectively) [3], [4]. Karaoz et al. only studied rat MSCs, and did not compare rat and human MSCs [5]. Martínez-Lorenzo et al. studied 18 MSC markers of humans and other species. However, many of these markers were only expressed by 10–70% of MSCs; therefore, it is unclear whether these markers are expressed by MSCs of these species [7].

Therefore, in this study, we used a large number of antibodies to determine whether a range of markers are present or absent on the surface of rabbit and human MSCs. Expression of each surface marker was studied on MSCs at passage 3 using flow cytometry, which were repeated four times. The mean percentage of human or rabbit MSCs that expressed the surface marker was determined. To definitively determine whether the protein was present or absent from the surface of human or rabbit MSCs, only antibodies that were advertised as being reactive against the human and/or rabbit surface markers were used. Therefore, we provided values (percentage of cells labeled by a marker) fulfilled or close to fulfilling Dominici's criteria to define MSCs as being positive or negative for the given markers.

## Materials and Methods

### Ethics statement

All animals were cared for in strict accordance with the recommendations of the Guide for the Care and Use of Laboratory Animals of the National Institutes of Health of the Republic of China (Taiwan). The protocol was approved by the Committee for Animal Experimentation of Chang Gung Memorial Hospital (permit number: 2010122206). All surgery was performed under general anesthesia, and all efforts were made to minimize animal suffering.

### Preparation of rabbit bone marrow MSCs

The femurs of New Zealand white rabbits (weight, ∼3.0 kg; age, 16–20 weeks) were harvested under general anesthesia and sterile conditions. Muscle and all connective tissue were detached from the femur. The ends of the bone were removed and an 18-gauge needle was inserted into the femoral shaft. The bone marrow of the shaft was extruded by flushing with low glucose Dulbecco's Modified Eagle Medium (DMEM-LG; Gibco-BRL, Carlsbad, CA) supplemented with 10% fetal bovine serum (FBS; Gibco-BRL), 100 U/ml penicillin (Hyclone, Logan, UT), and 100 µg/ml streptomycin (Hyclone). The bone marrow plug suspension was dispersed by pipetting, filtered through a 70 µm mesh nylon filter (Becton Dickinson Biosciences, Bedford, MA), and centrifuged at 400×g for 5 min. Pellets were resuspended in buffer (0.154 M NH_4_Cl, 10 mM KHCO_3_, and 0.1 mM EDTA) for 5 min to lyse red blood cells, and then centrifuged at 400×g for 5 min. The supernatant was decanted by pipetting. Cells (1×10^7^) were seeded into tissue culture plates (100 mm diameter) and incubated at 37°C in 5% CO_2_. After 4 days of incubation, non-adherent cells were removed by replacing the medium; thereafter, the medium was changed twice per week. When cells were 80–90% confluent, they were harvested by treatment with 0.05% trypsin-EDTA (Gibco) for 5–10 min at 37°C, and then centrifuged at 400×g for 5 min. Resuspended cells were re-plated at a density of 1.5×10^6^ cells per plate. The culture medium was changed twice per week.

### Examination of the differentiation potential of rabbit MSCs

When rabbit MSCs were at passage 3, their potential to differentiate into mesenchymal cells was examined.

For osteogenic differentiation, rabbit MSCs were cultured for 3 weeks in DMEM-LG containing 10% FBS, 50 µg/ml ascorbic acid (Sigma-Aldrich, St Louis, MO), 10 mM β-glycerophosphate (Calbiochem, San Diego, CA), and 100 nM dexamethasone (Sigma-Aldrich). Cells were rinsed with phosphate-buffered saline (PBS, pH 7.4), fixed with PBS (pH 7.4) containing 4% formaldehyde at room temperature for 10 min, and incubated with 2% Alizarin Red (pH 4.2, Sigma-Aldrich) at room temperature for 30 min to label calcium.

For chondrogenic differentiation, 2.5×10^5^ rabbit MSCs were placed into a 15 ml polypropylene tube (Falcon, Bedford, MA) and centrifuged. Pellets were cultured at 37°C and 5% CO_2_ in 1 ml high glucose Dulbecco's Modified Eagle Medium containing 1× insulin-transferrin-sodium selenite (Sigma-Aldrich), 40 µg/ml proline (Sigma-Aldrich), 100 µg/ml sodium pyruvate (Sigma-Aldrich), 50 µg/ml ascorbate-2-phosphate (Sigma-Aldrich), 10 ng/ml transforming growth factor-β1 (Peprotech, Rocky Hill, NJ), and 100 nM dexamethasone (Sigma-Aldrich). Half of the medium was replaced with fresh medium every 2–3 days for 3 weeks. Pellets were embedded in paraffin, cut into 4 µm-thick sections, and stained with Alcian blue solution (Sigma-Aldrich) for 30 min at room temperature to label sulfated cartilage glycosaminoglycans (GAGs).

For adipogenic differentiation, rabbit MSCs were cultured for 3 weeks in DMEM-LG containing 10% FBS, 0.5 mM isobutyl-methylxanthine (Sigma-Aldrich), 0.2 mM indomethacin (Sigma-Aldrich), 100 nM dexamethasone (Sigma-Aldrich), and 10 µg/mL insulin (Sigma-Aldrich). Cells were rinsed with PBS (pH 7.4), fixed with PBS (pH 7.4) containing 4% formaldehyde at room temperature for 10 min, and incubated with 0.5% Oil Red O solution (Sigma-Aldrich) for 30 min at room temperature to label neutral lipids.

### Detection of surface markers on rabbit MSCs

Rabbit MSCs at passage 3 were harvested and resuspended in culture medium at a density of 1×10^6^ cells/ml. The surface markers of these cells were studied using flow cytometry and 25 antibodies against rabbit surface antigens, namely, CD13, CD14, CD29, CD31, CD34, CD44, CD45, CD49d, CD49f, CD51, CD54, CD59, CD71, CD73, CD90, CD105, CD106, CD133, CD166, MHC I, MHC II, α-smooth muscle actin (α-SMA), cytokeratin, desmin, and vimentin. Cells were either only stained with the primary antibody, or with a primary and a secondary antibody. The staining method used was in accordance with the instruction leaflet that accompanied the antibody.

Rabbit MSCs were directly stained with the mouse anti-rabbit CD13, CD49f, CD54, CD59, CD71, CD105, vimentin (all from GeneTex, Irvine, CA), CD29 (Millipore, Temecula, CA), CD51 (Novus, Littleton, CO), CD73 (Abcam, Cambridge, MA), and cytokeratin (Thermo Fisher Scientific, Waltham, MA) monoclonal antibodies, and with the rabbit anti-rabbit CD31, CD49d, and CD133 (all from Bioss Inc., Wobum, MA) polyclonal antibodies. Cells (2×10^5^) were incubated with the appropriate antibody in 0.1 ml PBS containing 0.1% bovine serum albumin for 30 min at room temperature.

Secondary antibodies were used following staining of rabbit MSCs with mouse anti-rabbit CD14, CD44, CD45, MHC I, MHC II (all from Antigenix America, Huntington Station, NY), and α-SMA (AbD Serotec Ltd., Oxford, UK) monoclonal antibodies; with rat anti-rabbit CD34, desmin (all from GeneTex), and CD90 (Thermo Fisher Scientific) monoclonal antibodies; and with rabbit anti-rabbit CD106 and CD166 (both from Bioss Inc.) polyclonal antibodies. Cells (2×10^5^) were incubated with the appropriate antibody in 0.1 ml PBS containing 0.1% bovine serum albumin for 30 min at room temperature. Then, cells were stained with polyclonal secondary antibodies at room temperature for 30 min. The secondary antibodies used were fluorescein isothiocyanate (FITC)-conjugated rat anti-mouse IgG (eBioscience, San Diego, CA), Alexa Fluor 488-conjugated goat anti-rat IgG (Invitrogen, Carlsbad, CA), and FITC-conjugated goat anti-rabbit IgG (BD PharMingen, San Diego, CA).

Labeled cells were analyzed four times by performing flow cytometry. Prior to this analysis, samples were washed, and dead cells and cell debris were removed. Data were analyzed using Cell Quest software (Becton Dickinson Biosciences, Heidelberg, Germany) and the percentage of cells that were positive for a marker are shown as the mean ± standard deviation (n = 4).

### Preparation of human bone marrow MSCs

Human bone marrow MSCs were obtained from Lonza (catalogue number PT-2501, BioWhittaker Europe SPRL, Belgium). These cells were cultured in MSCGM culture medium (Lonza, Walkerville, MD) in a T-75 flask at 37°C in 5% CO_2_. When cells were 80–90% confluent, they were passaged by incubating with 0.25% trypsin and 1 mM EDTA (catalogue number CC-3232, Lonza, Walkerville, MD) for 3 min at 37°C. Cells at passage 3 were used for experiments.

### Examination of the differentiation potential of human MSCs

Human MSCs were induced to differentiate into cells of mesenchymal lineages and were subsequently stained as described for rabbit MSCs.

### Detection of surface markers on human MSCs

Human MSCs at passage 3 were harvested and resuspended in culture medium at a density of 1×10^6^ cells/ml. The surface markers of these cells were studied using flow cytometry and 25 antibodies for human surface antigens, namely, CD13, CD14, CD29, CD31, CD34, CD44, CD45, CD49d, CD49f, CD51, CD54, CD59, CD71, CD73, CD90, CD105, CD106, CD133, CD166, MHC I, MHC II, α-SMA, cytokeratin, desmin, and vimentin.

Human MSCs were directly stained with mouse anti-human CD13, CD49f, CD54, CD59, CD71, CD105, vimentin (all from GeneTex), CD29 (Millipore), CD51 (Novus), CD73 (Abcam), and cytokeratin (Thermo Fisher Scientific) monoclonal antibodies, and with rabbit anti-human CD31, CD49d, and CD133 (all from Bioss Inc.) polyclonal antibodies. Cells (2×10^5^) were incubated with the appropriate antibody in 0.1 ml PBS containing 0.1% bovine serum albumin for 30 min at room temperature.

Secondary antibodies were used following the staining of human MSCs with mouse anti-human CD14, CD44, CD45, CD90, CD166, MHC I, MHC II (all from BD PharMingen, San Diego, CA), desmin (GeneTex), and α-SMA (AbD Serotec Ltd.) monoclonal antibodies; the rat anti-human CD34 (GeneTex) monoclonal antibody; and the rabbit anti-human CD106 (Bioss Inc.) polyclonal antibody. Cells (2×10^5^) were incubated with the appropriate antibody in 0.1 ml PBS containing 0.1% bovine serum albumin for 30 min at room temperature. Then, cells were stained with polyclonal secondary antibodies as described for rabbit MSCs.

Labeled human MSCs were analyzed as described for rabbit MSCs.

### Additional study for detection of surface markers on rabbit adipose derived MSCs

As the current study was performed using rabbit bone marrow derived stem cells, we felt like to test the justifications of our methodology by an additional study that detected the surface markers on rabbit adipose tissue derived stem cells.

Adipose tissue cells were isolated from rabbit inguinal and interscapular adipose tissues. It was cut into small fragments and digested with 1 mg/ml collagenase I (Sigma Aldrich) under gentle shaking for 30 min at 37°C. Then DMEM-LG containing 10% FBS was added to neutralize the enzyme activity. The contents were resuspended in washing buffer (1X PBS) and were filtered with a 100 µm mesh nylon filter (Becton Dickinson Biosciences), washed via three centrifugations (800×g for 5 min at room temperature), and then resuspended in complete culture medium (DMEM-LG with 10% FBS). Cells were incubated at 37°C in a humidified atmosphere containing 5% CO_2_. After 24 hours plating, all nonadherent cells were removed. The examination of the differentiation potential and detection of surface markers of rabbit adipose tissue derived MSCs were performed by the same methodology for rabbit bone marrow MSCs.

### Additional study for RT-PCR analysis of CD44 and CD105 mRNA expression in rabbit and human MSCs

Total RNA was extracted from 10^5^–10^6^ human and rabbit MSCs by using Trizol (Invitrogen). cDNA was synthesized using SuperScript III First-Strand Synthesis System RT-PCR kit (Invitrogen), according to the manufacturer's instructions. Obtained cDNA served as a template for PCR with gene-specific primers as follows: rabbit CD44, forward (TCATCCTGGCATCCCTCTTG), reverse (CCGTTGCCATTGTTGATCAC); rabbit CD105, forward (TGACATACAGCACCAGCCAG), reverse (AGCTCTGACACCTCGTTTGG); rabbit β-actin, forward (GTGCTTCTAGGCGGACTGTTAGA), reverse (CACGAATAAAGCCATGCCAAT); human CD44, forward (CATAGAAGGGCACGTGGTGAT), reverse (ATACTGGGAGGTGTTGGATGTGA); human CD105, forward (CCTACGTGTCCTGGCTCATC), reverse (GGTGTGTCTGGGAGCTTGAA); human β-actin, forward (TGCCGACAGGATGCAGAAG), reverse (GCCGATCCACACGGAGTACT). The mRNA levels were evaluated using the comparative cycle threshold method with β-actin for normalization. PCR products were separated on a 2% agarose gel (Promega, Madison, WI).

## Results

### Preparation of rabbit MSCs

**Figure 1:**
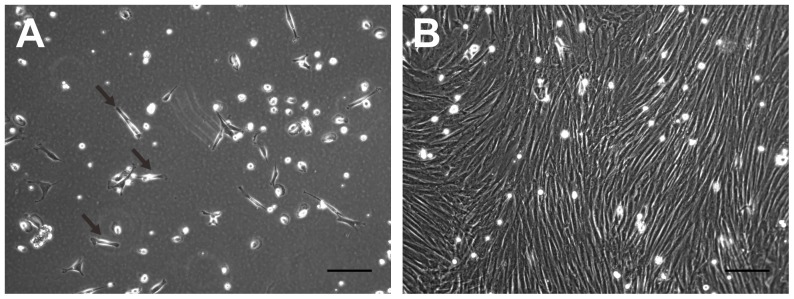
Morphology of rabbit MSCs observed using an inverted microscope. A. At 1 week after seeding, only a few fibroblast-like cells (arrows) adhere to the culture surface (original magnification, 100×; scale bar, 10 µm). B. At 2 weeks after seeding, a large number of fibroblast-like cells adhere to the culture surface (original magnification, 100×; scale bar, 10 µm).

At 1 week after plating rabbit MSCs, only a few adherent fibroblast-like cells were observed using an inverted microscope (Figure 1A). However, at 2 weeks after plating, a large number of fibroblast-like cells had adhered to the culture surface (Figure 1B). When adherent cells were 80–90% confluent, they were harvested for further analysis.

### Differentiation potential of rabbit MSCs

**Figure 2:**
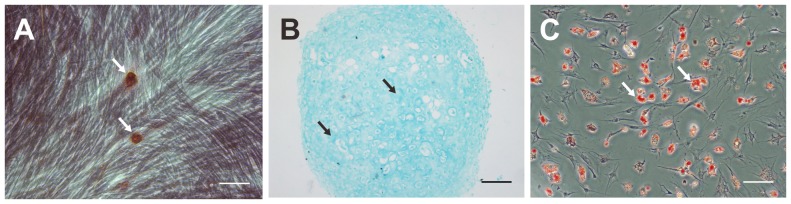
Rabbit MSCs observed using an inverted microscope after differentiation and staining. A. After osteogenic differentiation of rabbit MSCs and Alizarin red staining, calcium deposition is observed (arrows; original magnification, 100×; scale bar, 10 µm). B. After chondrogenic differentiation of rabbit MSCs and Alcian blue staining, sulfated cartilage GAGs are observed (arrows; original magnification, 100×; scale bar, 100 µm). C. After adipogenic differentiation of rabbit MSCs and Oil Red O staining, accumulation of intracellular neutral lipid vacuoles is observed (arrows; original magnification, 100×; scale bar, 10 µm).

After osteogenic differentiation of rabbit MSCs and Alizarin red staining, aggregates or nodules of calcium were observed (Figure 2A). After chondrogenic differentiation of rabbit MSCs and Alcian blue staining, sulfated cartilage GAGs, which are a component of articular cartilage, were detected (Figure 2B). After adipogenic differentiation of rabbit MSCs and Oil Red O staining, accumulation of intracellular neutral lipid vacuoles was detected (Figure 2C).

### Detection of surface markers of rabbit MSCs

Figure S1 shows flow cytometric analysis of rabbit MSCs labeled with 25 antibodies against various surface markers. The mean percentages of rabbit MSCs labeled with each of these 25 markers are shown in Table 1. Among these 25 mean values, 23 fulfilled or were close to fulfilling Dominici's criteria to define rabbit MSCs as being positive or negative for the given marker [1]. According to Dominici's criteria, a cell sample is positive for a marker if more than 95% of cells express the marker, whereas it is negative for a marker if less than 2% of cells express the marker [1]. Two mean values (12.99% for CD51 and 77.91% for desmin) were not close to fulfilling Dominici's criteria. This indicated that 12.99% of rabbit MSCs expressed CD51, whereas 77.91% expressed desmin. As the rabbit MSCs were heterogeneous and unavoidably contaminated with other cells, it would be reasonable to conclude that CD51 was absent from rabbit MSCs, whereas desmin was present on rabbit MSCs.

**Table 1:** Mean percentages of rabbit MSCs that were labeled with each of the 25 markers, as determined by flow cytometry.

Percentage of marker-labeled rabbit MSCs at P3	Mean±SD	Reactivity
CD13	0.49±0.23	–
CD14	2.13±0.77	–
CD29	2.84±0.23	–
CD31	2.78±1.12	–
CD34	2.12±0.79	–
CD44	97.32±1.32	+
CD45	1.71±0.25	–
CD49d	3.56±1.99	–
CD49f	1.37±0.47	–
CD51	12.99±1.42	–
CD54	1.02±1.15	–
CD59	1.65±0.44	–
CD71	0.74±0.22	–
CD73	0.73±0.14	–
CD90	1.48±0.84	–
CD105	1.14±0.39	–
CD106	1.00± 0.74	–
CD133	2.02±0.46	–
CD166	0.80 ± 0.30	–
MHC I	2.40±0.46	–
MHC II	1.66±0.21	–
α-SMA	95.10±2.00	+
Cytokeratin	1.64±0.41	–
Desmin	77.91±1.78	+
Vimentin	95.68±3.58	+

Among the 25 mean percentages, 23 fulfilled or were close to fulfilling Dominici's criteria to define rabbit MSCs as being positive or negative for the given marker*. Only two mean values (12.99% for CD51 and 77.91% for desmin) had slight deviation from Dominici's criteria. If CD51 was considered to be absent, while desmin present, our result show that CD13, CD14, CD29, CD31, CD34, CD45, CD49d, CD49f, CD51, CD54, CD59, CD71, CD73, CD90, CD105, CD106, CD133, CD166, MHC I, MHC II and cytokeratin are absent from rabbit MSCs, whereas CD44, α-SMA, vimentin and desmin are present on rabbit MSCs.

*Dominici's criteria: a cell sample is positive for a given marker if more than 95% of cells express the marker, whereas it is negative if less than 2% of cells express the marker [1].

The data showed that CD13, CD14, CD29, CD31, CD34, CD45, CD49d, CD49f, CD51, CD54, CD59, CD71, CD73, CD90, CD105, CD106, CD133, CD166, MHC I, MHC II and cytokeratin were absent from rabbit MSCs, whereas CD44, α-SMA, desmin and vimentin were present on rabbit MSCs.

### Preparation of human MSCs

**Figure 3:**
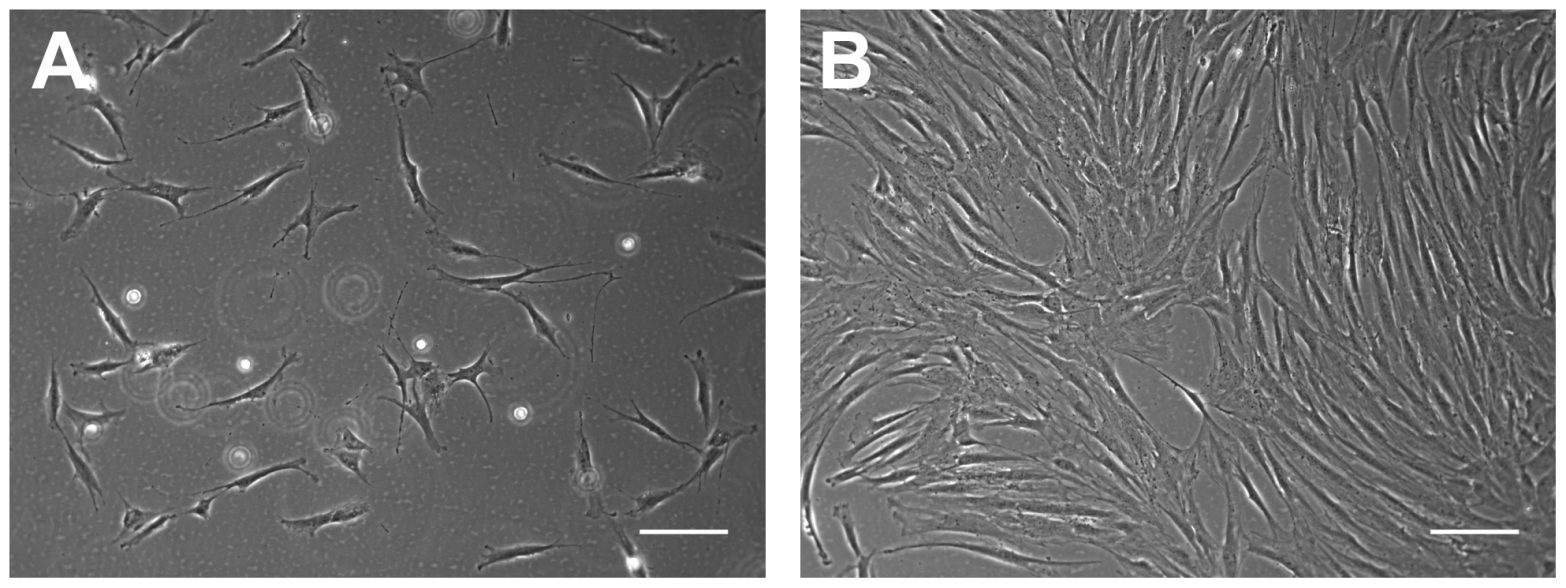
Morphology of human MSCs observed using an inverted microscope. A. At 1 day after seeding, only a few fibroblast-like cells are adhered to the culture surface (original magnification, 100×; scale bar, 10 µm). B. At 3–4 days after seeding, a large number of fibroblast-like cells are adhered to the culture surface (original magnification, 100×; scale bar, 10 µm).

At 1 day after plating of human MSCs, only a few adherent fibroblast-like cells were observed using an inverted microscope (Figure 3A). However, at 3–4 days after plating, a large number of fibroblast-like cells had adhered to the culture surface (Figure 3B). When adherent cells were 80–90% confluent, they were harvested for further analysis.

### Differentiation potential of human MSCs

**Figure 4:**
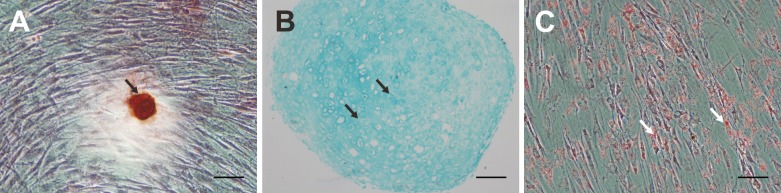
Human MSCs observed using an inverted microscope after differentiation and staining. A. After osteogenic differentiation of human MSCs and Alizarin red staining, calcium deposition is observed (arrow, original magnification, 100×; scale bar, 10 µm). B. After chondrogenic differentiation of human MSCs and Alcian blue staining, sulfated cartilage GAGs are observed (arrows; original magnification, 100×; scale bar, 100 µm). C. After adipogenic differentiation of human MSCs and Oil Red O staining, accumulation of intracellular neutral lipid vacuoles is observed (arrows; original magnification, 100×; scale bar, 10 µm).

After osteogenic differentiation of human MSCs and Alizarin red staining, calcium deposits were observed (Figure 4A). After chondrogenic differentiation of human MSCs and Alcian blue staining, sulfated cartilage GAGs were detected (Figure 4B). After adipogenic differentiation of human MSCs and Oil Red O staining, accumulation of intracellular neutral lipid vacuoles was detected (Figure 4C).

### Detection of surface markers of human MSCs

Figure S2 shows flow cytometric analysis of human MSCs labeled with 25 markers against various surface markers. The mean percentages of human MSCs labeled with each of these markers are shown in Table 2. Among these 25 mean values, 24 fulfilled or were close to fulfilling Dominici's criteria to define human MSCs as being positive or negative for a given marker [1]. One mean value (13.72% for CD71) was not close to fulfilling Dominici's criteria. This indicated that 13.72% of human MSCs expressed CD71. As human MSCs were heterogeneous and unavoidably contaminated with other cells, it would be reasonable to conclude that CD71 was absent from human MSCs.

**Table 2:** Mean percentages of human MSCs that are labeled with each of the 25 markers, as determined by flow cytometry.

Percentage of marker-labeled human MSCs at P3	Mean±SD	Reactivity
CD13	92.86±8.91	+
CD14	3.08±0.59	–
CD29	98.51±1.21	+
CD31	4.64±0.55	–
CD34	1.87±0.60	–
CD44	99.21±0.47	+
CD45	3.92±1.69	–
CD49d	5.44±1.26	–
CD49f	2.73±1.13	–
CD51	3.78±0.95	–
CD54	1.39±0.50	–
CD59	99.94±0.04	+
CD71	13.72±0.87	–
CD73	98.67±0.66	+
CD90	98.95±0.38	+
CD105	99.28±0.34	+
CD106	2.17±1.01	–
CD133	1.60±0.78	–
CD166	96.81±0.74	+
MHC I	98.63±0.25	+
MHC II	2.02±0.40	–
α-SMA	85.55±2.14	+
Cytokeratin	0.84±0.08	–
Desmin	6.17±1.59	–
Vimentin	98.54±0.29	+

Among the 25 mean percentages, 24 fulfilled or were close to fulfilling Dominici's criteria to define human MSCs as being positive or negative for a given marker*. Only one mean value (13.72% for CD71) had slight deviation from Dominici's criteria. If CD71 was considered to be absent, our result show that CD14, CD31, CD34, CD45, CD49d, CD49f, CD51, CD54, CD71 CD106, CD133, MHC II, cytokeratin and desmin are absent from human MSCs, whereas CD13, CD29, CD44, CD59, CD73, CD90, CD105, CD166, MHC I, α-SMA, and vimentin are present on human MSCs.

*Dominici's criteria: a cell sample is positive for a given marker if more than 95% of cells express the marker, whereas it is negative if less than 2% of cells express the marker [1].

Our results showed that CD14, CD31, CD34, CD45, CD49d, CD49f, CD51, CD54, CD71, CD106, CD133, MHC II, cytokeratin, and desmin were absent from human MSCs, whereas CD13, CD29, CD44, CD59, CD73, CD90, CD105, CD166, MHC I, α-SMA, and vimentin were present on human MSCs.

### Comparison of surface markers between human and rabbit MSCs

CD14, CD31, CD34, CD45, CD49d, CD49f, CD51, CD54, CD71, CD106, CD133, MHC II, and cytokeratin were absent from both rabbit and human MSCs, whereas CD44, α-SMA, and vimentin were present on both cell lines.

CD13, CD29, CD59, CD73, CD90, CD105, CD166, and MHC I were present on human MSCs, but not on rabbit MSCs. However, desmin was present on rabbit MSCs, but absent on human MSCs.

In summary, the surface expression of nine markers differed between human and rabbit MSCs, whereas the surface expression of 16 markers was the same in the two cell lines. The former 9 markers are generally found on mesenchymal cells, instead of hematopoietic cells.

### Additional study for detection of surface markers on rabbit adipose derived MSCs and comparison of surface markers between rabbit adipose and bone marrow derived MSCs

The results were shown in Table 3. It showed, of the 25 markers tested, all except CD51 and desmin were the same between rabbit adipose derived and bone marrow MSCs. That is 92% of the surface markers of the two cell lines are the same. The similarity of the surface markers of these two cell lines provided the justifications of our observations that the surface markers between human and rabbit MSCs are indeed different.

**Table 3:** Mean percentages of rabbit adipose derived stem cells that were labeled with each of the 25 markers, as determined by flow cytometry.

Percentage of marker-labeled rabbit ASCs at P3	Mean±SD	Reactivity
CD13	0.75±0.57	–
CD14	0.90±0.62	–
CD29	1.47±0.86	–
CD31	1.40±0.29	–
CD34	1.53±0.49	–
CD44	96.97±0.58	+
CD45	0.85±0.35	–
CD49d	3.59±1.14	–
CD49f	0.50±0.15	–
CD51	85.38±7.96	+
CD54	0.58±0.14	–
CD59	0.64±0.19	–
CD71	0.80±0.27	–
CD73	0.52±0.21	–
CD90	1.17±0.41	–
CD105	1.64±0.64	–
CD106	0.69±0.38	–
CD133	1.24±0.41	–
CD166	0.90±0.69	–
MHC I	1.91±0.58	–
MHC II	1.32±1.12	–
α-SMA	90.61±2.20	+
Cytokeratin	0.48±0.21	–
Desmin	31.32±4.83	–
Vimentin	95.78±1.29	+

### Additional study for RT-PCR analysis of CD44 and CD105 mRNA expression in rabbit and human MSCs

**Figure 5:**
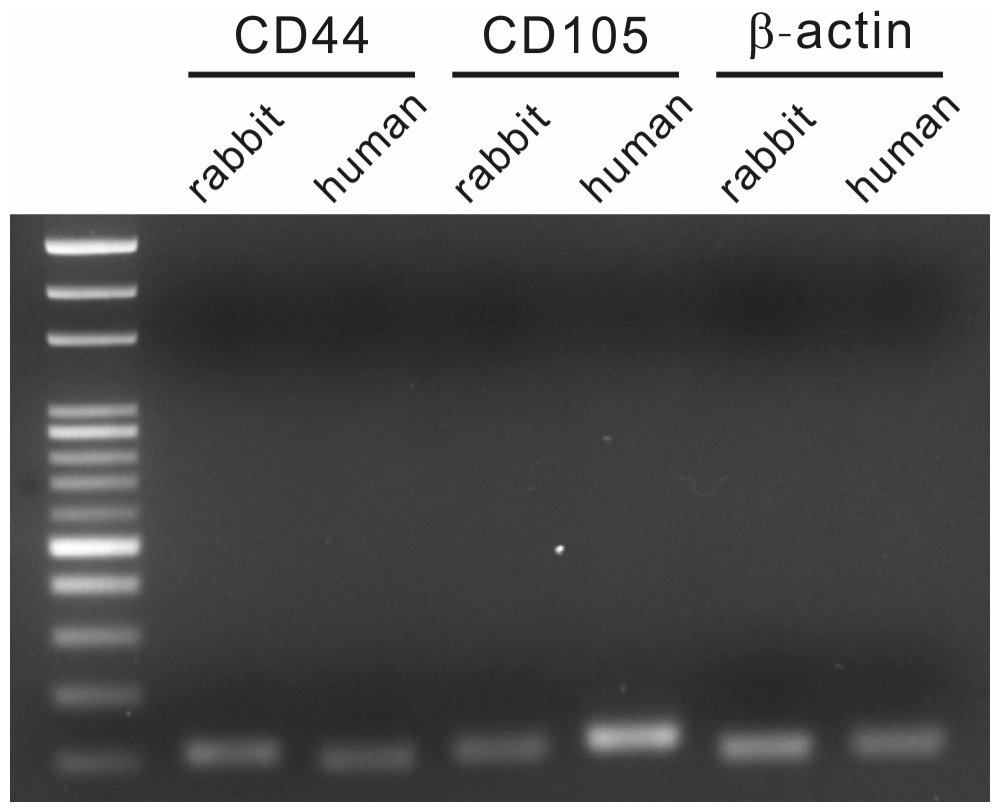
RT-PCR analysis of CD44 and CD105 mRNA expression in rabbit and human MSCs. The RT-PCR products from rabbit and human MSCs were normalized to the expression of β-actin. The electrophoresis of the rabbit MSCs showed a normalized expression of CD44 mRNA of 0.8, and CD105 mRNA of 0.59. The electrophoresis of the human MSCs showed a normalized expression of CD44 mRNA of 0.71, and CD105 mRNA of 2.4. The results indicated that the ratio between rabbit and human CD44 expression was 1∶0.89, while the ratio between rabbit and human CD105 expression was 1∶4.08. These results indicated that rabbit and human MSCs had similar expression of CD44 at the mRNA level. However, human MSCs, compared with rabbit MSCs, had apparent more expression of CD105 at the mRNA level.

The band density was digitalized by image analysis software (ImageJ) (Figure 5). The electrophoresis (RT-PCR) of the rabbit MSCs showed a normalized expression of CD44 mRNA of 0.8, and CD105 mRNA of 0.59. The electrophoresis of the human MSCs showed a normalized expression of CD44 mRNA of 0.71, and CD105 mRNA of 2.4. The results indicated that the ratio between rabbit and human CD44 expression was 1∶0.89, while the ratio between rabbit and human CD105 expression was 1∶4.08. Therefore, rabbit and human MSCs had similar expression of CD44 at the mRNA level. However, human MSCs, compared with rabbit MSCs, had apparent more expression of CD105 at the mRNA level. These findings were compatible with the results of the current study using flow cytometry.

## Discussion

The phenotype of human MSCs has been well documented; therefore, we first compared the phenotype of our human MSCs with that of previous studies to verify that our methodology was correct. Our human MSCs had the characteristic phenotypes of MSCs as they were positive for CD13, CD29, CD44, CD73, CD90, CD105, and CD166, but negative for CD14, CD31, CD34, CD45, and CD133. These phenotypes are considered to be important indicators of MSCs [1], [2], [4], [7].

However, our human MSCs were negative for an important marker, CD106, which is generally considered to be expressed by human MSCs [2]. In a systematic review of the cell surface markers of adult human MSCs, Mafi et al. found that some studies reported human adult MSCs to be CD106-positive, whereas other studies found them to be CD106-negative [8]. The proliferative stage and origin of the cells are important factors that affect surface marker expression [9], [10]. Halfon et al. reported that CD106 is downregulated in MSCs at passage 6 compared to those at passage 2 [9]. Gronthos et al. reported that the expression of surface antigens differs between adipose tissue-derived stromal cells and bone marrow-derived stromal cells [10]. Therefore, the negative CD106 reactivity of human bone marrow MSCs in the current study does not necessarily indicate that these cells are not actually MSCs. Therefore, all the surface marker data of our human MSCs was compared to that of the rabbit MSCs.

Given that the expression of surface markers on MSCs can be affected by many factors, as described above, we studied rabbit and human MSCs from the same origin (bone marrow), at the same passage (passage 3), with the same culture conditions, in the same laboratory, and using the same personnel to ensure that the results were convincing. In addition, the same methods were used to detect surface markers in both cell lines.

As mentioned above, our human MSCs had the characteristic phenotypes of MSCs. That is, our data showed the surface markers of our human stem cells were comparable to other existing studies. However, we have demonstrated differences of phenotypes between our human and rabbit stem cells. That is, the surface markers of our rabbit stem cells were different from the general expectations.

Although some studies reported MSCs to be simply positive or negative for specific markers [2]–[5], the definition of whether a cell exhibits positive or negative reactivity for a given marker is not unified. Flow cytometry is widely used, meaning it is important to specify the percentage of cells that are labeled by a marker, rather than simply stating that reactivity is positive or negative. The study by Martínez-Lorenzo et al. was one of the few that reported the percentage of marker-labeled cells [7]. However, some of their data were confusing. For instance, they found that 98% of human MSCs and 40% of rabbit MSCs expressed CD90 [7], meaning it is uncertain whether rabbit MSCs are positive or negative for CD90. Therefore, we were careful to avoid contaminating MSCs with other cell types, and only used antibodies that were marketed as being reactive against human and/or rabbit surface markers. In this way, we hoped that our data would fulfill (or be close to fulfilling) Dominici's criteria. For example, 1.48% of rabbit MSCs expressed CD90, fulfilling Dominici's definition of negative reactivity. Accordingly, we can conclude that rabbit MSCs do not express CD90.

We are not the first to report that the phenotypes of human and rabbit MSCs differ. Human MSCs are generally positive for CD90 [1], [2], [4], [11], [12], whereas rabbit MSCs are not [4]. We are also not the first to report the percentage of marker-labeled MSCs using flow cytometry [7]. However, we are the first to examine a large number of markers by reporting the percentages of marker-labeled MSCs, and most of our data fulfilled or were close to fulfilling Dominici's criteria of whether a cell sample is positive or negative for a given marker. This may be attributed to our careful isolation of MSCs to avoid contamination with other cell types and our careful selection of antibodies that were reactive against human and/or rabbit MSC markers. We did not label rabbit MSCs with an antibody that was only marketed as being reactive against the human protein, or vice versa. In this way, we could clearly determine the presence or absence of a given marker on human and rabbit MSCs.

For CD51 and desmin in rabbit MSCs and CD71 in human MSCs, the percentage of marker-labeled MSCs did not meet Dominici's criteria (≤2% or ≥95%) [1]. Specifically, 12.99% of rabbit MSCs expressed CD51, 77.91% of rabbit MSCs expressed desmin, and 13.72% of human MSCs expressed CD71. The rabbit and human MSCs used in the current study were isolated from a bone marrow mononuclear cell fraction based on their adherence to a plastic surface; therefore, these cells were essentially heterogeneous. Kassen and Gronthos et al. reported that such MSCs can be unavoidably contaminated with hematopoietic cells or other mononuclear cells [13], [14]. However, the data of the aforementioned three markers did not markedly differ from the levels stipulated by Dominici's criteria, and cells could be easily defined as positive or negative for each of the other markers.

We compared the data of the current study to that of four previous studies that reported the phenotypes of MSCs from different species (Table 4). The studies of Pittenger et al. and Lapi et al. did not specify how MSCs were defined as being positive or negative for a given marker [2], [4]. Only Dominici's study strictly defined the positive or negative reactivity of MSCs for a given marker according to the percentage of marker-labeled MSCs (≥95% cells labeled by the marker indicates positive reactivity, ≤2% cells labeled by the marker indicates negative reactivity) [1]. However, in this previous study, only eight markers were examined. The study by Martínez-Lorenzo et al. reported the percentages of MSCs that were labeled with a series of markers [7]. However, for many of these markers, only 10–70% of MSCs were labeled, creating confusion as to whether the MSCs are positive or negative for these markers.

**Table 4:** Comparison of data from the current study with data from four previous studies that examined the phenotypes of human and rabbit MSCs.

Authors	Pittenger et al. [2]	Dominich et al. [1]	Martinez-Lorenzo et al. [6]	Martinez-Lorenzo et al. [6]	Lapi et al. [4]	Lapi et al. [4]	Lee et al.	Lee et al.
Description of marker expression of MSCs	Positive or negative	Positive or negative*	%	%	Positive or negative	Positive or negative	%	%
MSCs source	Human	Human	Human	Rabbit	Human	Rabbit	Human	Rabbit
Passage	2	No mention	1	1	3	3	3	3
CD13			98.5±1.06	65.3±24.42			92.86±8.91	0.49±0.23
CD14	–	–			–	–	3.08±0.59	2.13±0.77
CD29	**+**				+	+	98.51±1.21	2.84±0.23
CD31	–		1.7±1.5	8.7±7.91			4.64±0.55	2.78±1.12
CD34	–	–	13±1.65	0.5±0.41			1.87±0.60	2.12±0.79
CD44	**+**		97.9±1.16	38.3±19.28	+	+	99.21±0.47	97.32±1.32
CD45	–	–			–	–	3.92±1.69	1.71±0.25
CD49d		–	85±9.92	5.3±5.06			5.44±1.26	3.56±1.99
CD49f			41.2±14.62	84.2±4.05			2.73±1.13	1.37±0.47
CD51	**+**						3.78±0.95	12.99±1.42
CD54	**+**		58.7±16.6	25.6±8.64			1.39±0.50	1.02±1.15
CD59			98.1±0.87	55±16.12			99.94±0.04	1.65±0.44
CD71	**+**		75.7±5.02	2.8±1.56			13.72±0.87	0.74±0.22
CD73		+	99.3±0.7	1.6±0.98			98.67±0.66	0.73±0.14
CD90	**+**	+	98±0.85	40.1±15.47	+	–	98.95±0.38	1.48±0.84
CD105	**+**	+	95±0.92	20.5±11.44			99.28±0.34	1.14±0.39
CD106	**+**		0.2±0.33	3.2±2.02			2.17±1.01	1.00±0.74
CD133			1.2±0.8	13.5±7.44			1.60±0.78	2.02±0.46
CD166	**+**		98±1.49	38.7±5.49			96.81±0.74	0.80±0.30
MHC I					–	–	98.63±0.25	2.40±0.46
MHC II		–	30.2±13	21.5±2.83	–	–	2.02±0.40	1.66±0.21
α-SMA					+	+	85.55±2.14	95.10±2.00
Cytokeratin					–	–	0.84±0.08	1.64±0.41
Desmin					+	+	6.17±1.59	77.91±1.78
Vimentin					+	+	98.54±0.29	95.68±3.58

The studies of Pittenger et al. and Lapi et al. did not specify how MSCs were defined as positive or negative for a given marker. Only Dominici's study strictly defined the positive or negative reactivity of MSCs for a given marker according to the percentage of marker-labeled cells^§^; however, only a small number of markers was studied. The study by Martínez-Lorenzo et al. reported the percentage of MSCs that were labeled with a marker; however, many of their results did not fulfill Dominici's criteria^§^.

§ Dominici's criteria: a cell sample is positive for a given marker if more than 95% of cells express the marker, whereas it is negative if less than 2% of cells express the marker [1].

*: ≥95% of cells labeled by the marker (+), ≤2% of cells labeled by the marker (-).

We also studied the expression of 11 markers in human MSCs at passage 6. Of these markers, most were slightly downregulated in human MSCs at passage 6 compared with those at passage 3 (Table 5); however, these differences were not statistically significant. These findings are in agreement with those of Halfon [9]. In human MSCs at passage 3, the data of 8 of the 11 markers fulfilled Dominici's criteria, whereas at passage 6 this was only true of four markers.

**Table 5:** Mean percentages of human MSCs that were labeled with each of 11 markers at passage 3 and 6.

Marker	P3	P6
CD13	92.86±8.91	88.74±5.95
CD14	3.08±0.59	3.08±0.68
CD29	98.51±1.21	94.20±3.06
CD34	1.87±0.60	0.21±0.07
CD44	99.21±0.47	95.18±3.40
CD45	3.92±1.69	2.22±0.30
CD73	98.67±0.66	93.49±3.22
CD90	98.95±0.38	91.35±5.74
CD105	99.28±0.34	82.90±2.68
CD133	1.60±0.78	0.19±0.08
CD166	96.81±0.74	79.35±14.64

Ten of the eleven markers were slightly downregulated in human MSCs at passage 6 compared with those at passage 3.

In conclusion, this study reports the percentages of human and rabbit MSCs that were labeled with 25 surface markers. Most of the data fulfilled or were close to fulfilling Dominici's criteria, meaning there was no ambiguity over whether a given marker was present or absent from the surface of human or rabbit MSCs. In total, the surface expression of nine markers differed between human and rabbit MSCs, whereas the surface expression of 16 markers was the same in the two cell lines. This study shows that there are marked differences between the surface markers of human MSCs and those of rabbit MSCs.

Finally, it is the general assumption that MSCs have the potential to differentiate into cells of mesenchymal lineages that are characterized by the presence of particular marker proteins on their surfaces, however, it should be noted that the surface markers between human and rabbit MSCs are different; CD73, CD90 and CD105 are examples of surface molecules generally used to identify human MSCs that are not expressed on rabbit MSCs. The current study stresses the importance of not introducing the surface markers of human MSCs to characterize the surface markers of the MSCs of other species. For example, lack of CD73, CD90 or CD105 does not exclude the possibility of stem cells of other species. We hope our data will help to clear the confusion of others when they are examining the surface markers to characterize the MSCs of a certain species.

As those studies described above were performed using bone marrow derived stem cells, we have confirmed these results by testing our methodology on rabbit adipose tissue derived stem cells. This additional study showed similarity of surface expression between rabbit bone marrow and adipose tissue derived stem cells because, of the 25 markers, all except CD51 and desmin (92%) were the same between the two cell lines. This observation provided a biological insight that the phenotypes of rabbit adipose tissue-derived and rabbit bone marrow-derived stem cells are similar, although not completely identical. It further provided the justifications that the surface markers of rabbit MSCs are indeed different from those of human MSCs.

Some recent reports introduced both flow cytometry and RT-PCR to detect MSC surface markers [15]. Therefore, in addition to flow cytometry, we also performed RT-PCR for detection of two common surface markers of our rabbit and human MSCs. The results showed rabbit and human MSCs had similar expression of CD44 at the mRNA level. However, human MSCs, compared with rabbit MSCs, had apparent more expression of CD105 at the mRNA level. These findings were compatible with the results of the current study using flow cytometry to detect MSC surface markers. In the flow study, human MSCs were positive for CD44 and CD105, while rabbit MSCs were positive for CD44, but negative for CD105.

However, Screven et al. has demonstrated that gene expression at the transcriptional level does not always translate to the protein level in a specific cell or tissue type [15]. Therefore, we are not sure the detection of other markers using flow cytometry and RT-PCR will have the same results. An extensive study of human and rabbit MSC surface markers using RT-PCR is mandatory in the future.

## Supporting Information

Figure S1 **Flow cytometry of rabbit MSCs.** Figure S1 shows flow cytometric analysis of rabbit MSCs labeled with 25 antibodies against various surface markers. The mean percentages of rabbit MSCs labeled with each of these 25 markers are shown in Table 1.
(TIF)

Figure S2 **Flow cytometry of human MSCs.** Figure S2 shows flow cytometric analysis of human MSCs labeled with 25 markers against various surface markers. The mean percentages of human MSCs labeled with each of these markers are shown in Table 2.
(TIF)
