# Supplementary figures and images for: Comparison of Surface Markers between Human and Rabbit Mesenchymal Stem Cells
Source: PLoS One. 2014 Nov 7;9(11):e111390. doi: 10.1371/journal.pone.0111390 (PMC4224397; doi:10.1371/journal.pone.0111390)

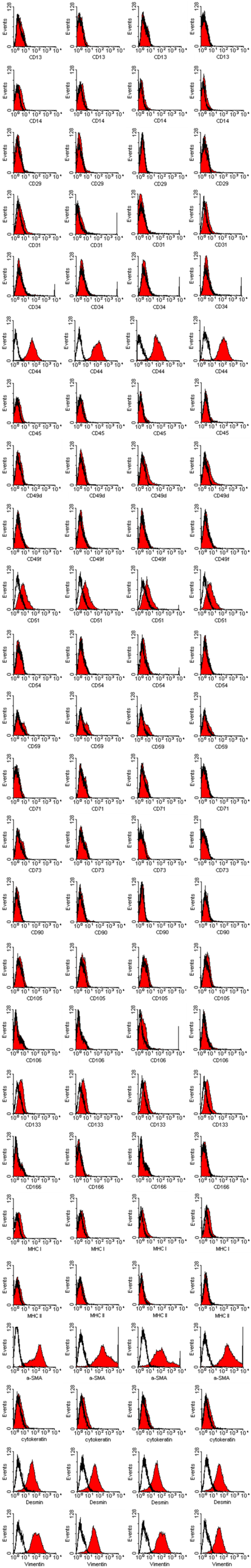

Supplement: Figure S1 — Flow cytometry of rabbit MSCs. Figure S1 shows flow cytometric analysis of rabbit MSCs labeled with 25 antibodies against various surface markers. The mean percentages of rabbit MSCs labeled with each of these 25 markers are shown in Table 1. (TIF) [file pone.0111390.s001.tif]

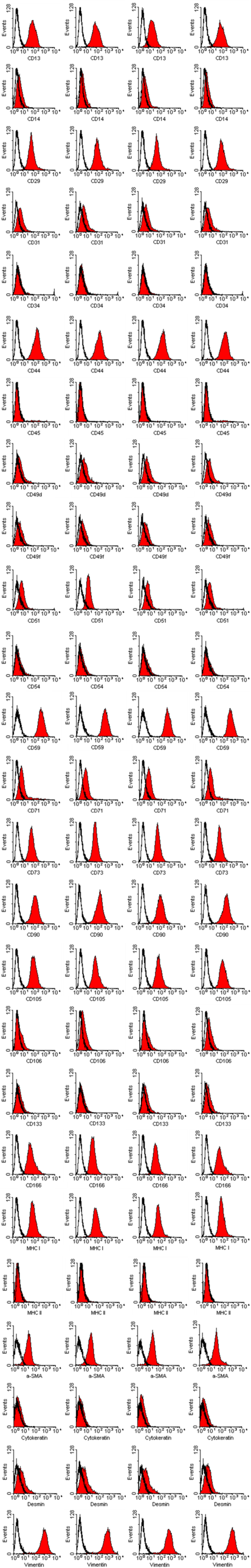

Supplement: Figure S2 — Flow cytometry of human MSCs. Figure S2 shows flow cytometric analysis of human MSCs labeled with 25 markers against various surface markers. The mean percentages of human MSCs labeled with each of these markers are shown in Table 2. (TIF) [file pone.0111390.s002.tif]
